# Case Report: Severe Anemia Associated With an Abomasal Fibrosarcoma in a Goat

**DOI:** 10.3389/fvets.2022.869017

**Published:** 2022-04-28

**Authors:** Matthias Gerhard Wagener, Georg Beythien, Markus Sterneberg, Antje Polifka, Thekla Großmann, Wolfgang Baumgärtner, Martin Ganter

**Affiliations:** ^1^Clinic for Swine, Small Ruminants, Forensic Medicine and Ambulatory Service, University of Veterinary Medicine Hannover, Foundation, Hannover, Germany; ^2^Department of Pathology, University of Veterinary Medicine Hannover, Foundation, Hannover, Germany

**Keywords:** hematocrit, melena, blood transfusion, leiomyoma, blood loss, neoplasia, small ruminants

## Abstract

A 10-year-old female goat was presented to the clinic with lethargy, emaciation, and pale mucous membranes. Laboratory diagnosis revealed severe anemia with regenerative character as well as melena. Blood transfusions were administered, but the animal's condition continued to deteriorate, so it was euthanized. The main finding in the necropsy was an abomasal neoplasia with two metastases in the mesenterium which was positive for vimentin, but negative for smooth muscle actin and c-kit using immunohistochemistry, indicating a fibrosarcoma that might have contributed to gastrointestinal blood loss. Further pathological findings consisted of changes in the liver cells as well as a cervical leiomyoma. These findings illustrate that intestinal blood loss due to neoplasia should also be considered in older goats with anemia.

## Introduction

Anemia is a common condition in small ruminants presented to the veterinary clinic, mainly caused by endoparasites, especially *Haemonchus contortus* (1). However, there are many other causes that can lead to anemia in goats. These include blood loss, hemolysis, or a decreased production of red blood cells (2). Blood loss can be caused by trauma such as dog bites, but also by bleeding wounds after castration or dehorning (2). Gastric ulcers, which can lead to gastrointestinal bleeding, have so far only been described very rarely in goats (3, 4). Anemia caused by haemonchosis can also be assigned as anemia due to blood loss. Since blood loss per adult worm can be up to 50 μL of blood per day, dramatic blood loss can occur in the event of a severe worm infestation (5). Worm management is therefore of great importance in small ruminant husbandry. Besides an adequate pasture management and the use of anthelmintics, there is also natural resistance to *Haemonchus contortus* in some animals (1, 6). Another endoparasite that is often associated with anemia in small ruminants is *Fasciola hepatica* that causes blood losses by tissue damage to the liver (7–10). In addition to endoparasites, severe infestation with ectoparasites such as lice or ticks can also lead to anemia (11, 12). Ticks also play a role, primarily as a vector for different pathogens. Some vector-borne diseases are hemoparasites for example *Anaplasma ovis, Babesia spp*., or *Mycoplasma ovis*, leading to lysis of red blood cells (13–16). Hemolysis can also result from the ingestion of large amounts of water, where the erythrocytes of goats generally have a higher osmotic resistance than those of sheep (17, 18). In sheep, hemolysis is also frequently observed in connection with copper poisoning, which can also occur in goats, which are less susceptible to it (19–22). Not only the oversupply, but also deficiencies of trace elements or vitamins can lead to anemic conditions in small ruminants, for example, as a consequence of iron (23, 24), copper (25, 26) or cobalamin (27) deficiencies. In those cases, the animals develop non-regenerative anemia due to a decreased production of red blood cells. In contrast, anemia caused by blood loss or hemolysis is characterized by increased erythropoiesis (23). Furthermore, neoplasms can be associated with anemia, there are reports that goats suffering from myelofibrosis (28), melanoma (29, 30) or thymoma (31) also revealed anemia.

This case report presents the clinical, laboratory, pathological and histological findings of a rare abomasal fibrosarcoma associated with severe regenerative anemia in a goat.

## Case Description

On 6/28/2021 (day 0), a 10-year-old German fawn goat, a traditional German goat breed, was presented to the Clinic for Swine, Small Ruminants, Forensic Medicine and Ambulatory Service, University of Veterinary Medicine Hannover, Foundation, Hannover, Germany. The goat came from a small herd of four female goats and three female sheep. The animals grazed in a landscape conservation area adjacent to a forest; a mineral supplement was provided. Vaccinations did not take place in this herd; worm treatments had been administered regularly in the past. The last routine deworming had taken place in April 2021 with fenbendazole. The owners of the goat reported that the animal had shown lethargy since the beginning of May. A week prior to admission to the clinic, the owners had also noticed emaciation and weakness. Three days before admission to the clinic, the goat had been pretreated with an antibiotic and fenbendazole by the local veterinarian. Thereby, the veterinarian had noticed a solid circumferential mass in the area of the right paralumbar fossa.

### Clinical Examination

**Figure 1:**
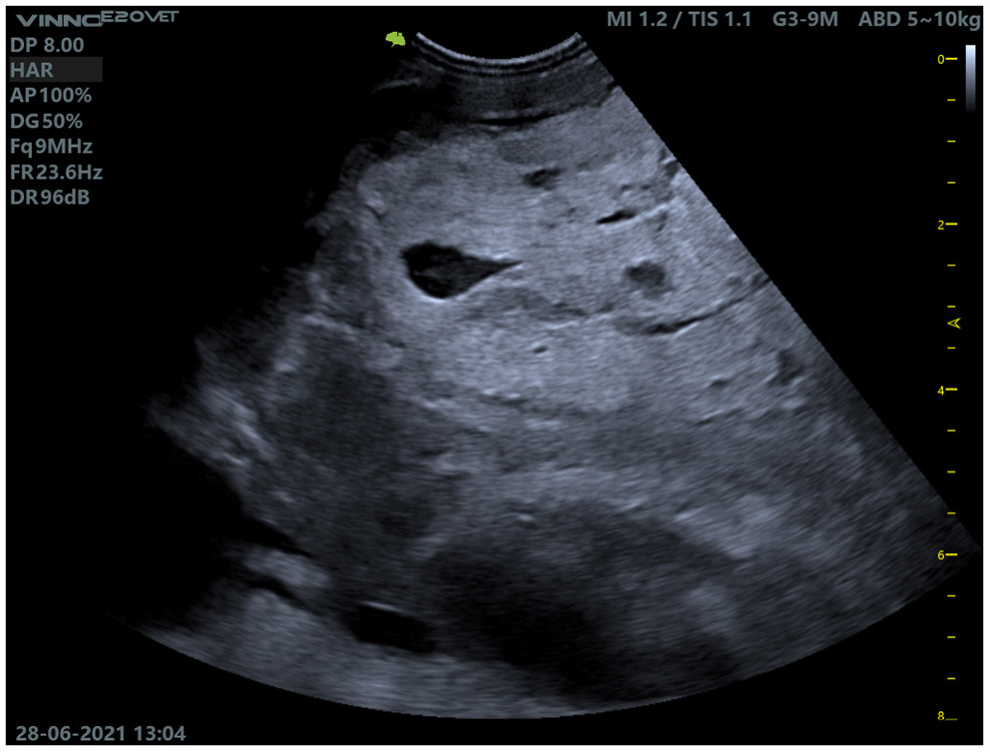
Ultrasound examination of the mass in the right paralumbar fossa. The mass was demarcated to the liver (not shown in the picture) and contained individual cavities.

During the clinical examination in the clinic, the goat was apathetic in sternal recumbency and was unable to keep its head up independently. The animal tried to stand up several times, but repeatedly collapsed. The animal was in a severely emaciated state, the protuberances of the spine and ribs were easily palpable. Bodyweight was 59.5 kg, rectal temperature 39.2°C. The conjunctival mucous membranes were white [FAMACHA©-score of 5 (32, 33)], indicating severe anemia. The episcleral vessels were slightly filled. Auscultation of the heart revealed tachycardia (heart rate of 144 beats/min). The heart sounds were regular but poorly separated, there were no secondary murmurs. Auscultation of the lungs revealed a weak inspiratory sound over the entire lung field and tachypnea (respiratory rate of 96/min). The goat's rumen was filled and fluctuating, two contractions of the rumen could be auscultated in 2 min in the left paralumbar fossa. In the right paralumbar fossa, an intra-abdominal slightly movable solid mass of about 15 cm was palpable. Palpation of the lymph nodes (*Lnn. mandibulares, Lnn. parotidei, Lnn. retropharyngei, Lnn. cervicalis superficiales, Lnn. subiliaci*) did not reveal any deviations from the norm. The skin of the goat was dry and scaly without macroscopic evidence of ectoparasites. The udder of the animal was asymmetrical, the left half of the udder was slightly larger than the right half of the udder and of coarse consistency, no secrete could be milked. Palpation of the limbs did not reveal any abnormalities. The mass in the right paralumbar fossa was further examined by ultrasound, it was well-demarcated against the liver, the major part was hyperechogenic with single anechogenic cavities (Figure 1).

### Laboratory Results

Blood samples (EDTA, Monovette 9 mL K3E, Lithium-Heparin, Monovette 9 mL LH and serum, Monovette 9 mL Z; all from Sarstedt AG & Co. KG, Nümbrecht, Germany) were taken from the jugular vein of the animal. A fecal sample was collected from the floor immediately after defecation. Blood and fecal samples were processed in the clinic's laboratory using the previously described routine methods (34). A detailed overview of the results of hematology and biochemistry is provided in Table 1.

**Table 1:** Hematology and biochemistry of the goat.

**Parameter**	**Unit**	**References**	**day 0**	**day 1**	**day 3**
Hemoglobin	g/L	90–121	21	32	28
PCV	L/L	0.24–0.35	0.06	0.09	0.08
MCHC	g/L	320–380	350	356	350
Platelets	G/L	350–650^#^	33	72	92
Leucocytes	G/L	7.8–19.6	20.8	23.1	20.1
Lymphocytes	G/L	3.5–11.9	3.2	1.9	2.3
PMN	G/L	2.6–9.9	16.1	20.3	15.6
Band neutrophils	G/L		0.2	0.2	1.7
Eosinophils	G/L	0–0.7	0.2	0.4	0.3
Basophils	G/L	0–0.3	0.2	0	0
Monocytes	G/L	0–0.5	0.8	0.4	0.2
Reticulocytes	‰		147	95	70
Anisocytosis			+++	+++	+++
Polychromasia			++	++	+++
Poikilocytosis			++	++	+++
CK	U/L	96–268	105		89
ASAT	U/L	66–135	51		70
GLDH	U/L	3.1–19.8	75		16
AP	U/L	31–1,941	113		107
GGT	U/L	26–63	42		
Protein (total)	g/L	59–82	56.8		50.8
Albumin	g/L	29–40	24.8		26.2
Glucose	mmol/L	2.3–3.6	8.9		
Bilirubin (total)	μmol/L	0.4–2.2	5.4		2.5
Creatinine	μmol/L	39–67	76		61
Urea	mmol/L	2.8–7.2	16.1		13
Calcium	mmol/L	2.2–2.7	1.9		1.5
Magnesium	mmol/L	0.9–1.4	0.7		0.5
Phosphate	mmol/L	1.2–2.9	1.7		1.7
Sodium	mmol/L	147–157	143.4		138.8
Potassium	mmol/L	4.0–5.8	4.5		4.4
Iron	μmol/L	11.6–38.1	9.2		4.6
Copper	μmol/L	7.0–24^*^	16.5		
Selenium	μg/L	80–500^*^	119.3		

*Reference values according to (35), except for platelets*

# *(36) or for*

* copper and selenium^*^ (37).

The hematological examination of the initial blood sample revealed severe anemia with a PCV (packed cell volume) of 0.06 L/L, but also reticulocytes and nucleated red blood cells, indication of a regenerative character. Further leucocytosis, lymphopenia, granulocytosis, and monocytosis were present. Additionally, a decreased numbers of platelets indicated thrombocytopenia. However, as some aggregated platelet were also detected in the blood smear, values must be interpreted carefully. Erythrocytes in the blood smear showed severe anisocytosis, polychromasia, and poikilocytosis. Furthermore, some erythrocytes revealed basophilic stippling. Plasma activity for GLDH (Glutamate dehydrogenase) was increased. Additionally, hypoproteinemia, hypoalbuminemia, hyperglycemia, hyperbilirubinemia, azotemia, hypocalcemia, hypomagnesemia, hyponatremia, and decreased iron levels were also observed in the plasma.

In the fecal sample of the animal, a low infestation with coccidia as well as gastrointestinal nematodes were detected after applying the sedimentation and flotation method with saturated sodium chloride solution. A test for melena (34) was positive.

### Treatment and Further Development

A venous catheter was inserted in the goat's jugular vein. Due to leucocytosis and the animal's very poor general condition, 10 mg/kg bodyweight (BW) amoxicillin (Amoxisel-Trockensubstanz 100 mg/mL, Selectavet Dr. Otto Fischer GmbH, Weyarn/Holzolling, Germany) and 0.27 mg/kg BW dexamethasone (Dexamethason 4 mg/mL, Bela-Pharm GmbH & Co. KG, Vechta, Germany) were administered intravenously. Due to calcium deficiency, 10 mL of a calcium solution was administered systemically (Calcitat N25, aniMedica GmbH, Senden-Bösensell, Germany). Additionally, 20 mL of an energy-containing nutrient containing Sodium propionate (Ceto Phyton®, Vetoquinol, Lure, France) was administered orally.

Due to the life-threatening anemia, a blood transfusion was administered through the venous catheter in the jugular vein. For this purpose, 500 mL of whole blood was collected from the jugular vein of a healthy donor animal and transferred into a blood bag (Single Blood bag-CPDA1-500 mL-16 G needle, Fioniavet, Fredericia, Denmark). The first few mL of the blood donation were administered very quickly to detect possible side effects; since no side effects were obvious after 10 min, the remaining blood donation was administered over a period of about 2 h. After the blood transfusion, the goat's condition improved significantly, the animal stood up and started eating hay.

On the following day (day 1), the animal was less apathetic than at the initial examination, could stand on its own and lift its head. The conjunctives showed a slightly red coloration. Auscultation of the lungs and heart showed similar findings as the previous day (respiratory rate: 92/min; heart rate: 140/min), the body temperature of the animal was 38.9°C. Since there was only a slight increase in PCV from 0.06 L/L to 0.09 L/L (Table 1), a second blood transfusion with 500 mL whole blood was performed. As gastrointestinal nematodes had been previously detected in the fecal sample, deworming was performed by administering 0.4 mg/kg BW moxidectin (Cydectin 0.1%, Zoetis Deutschland GmbH, Berlin, Germany) orally. Deworming was performed only after the second blood transfusion in order to stabilize the animal first by the blood transfusion, since deworming represents an additional stress in very emaciated and anemic animals.

The treatment with amoxicillin and propionate was continued on the following days. Both were administered twice daily, in the morning and in the evening. In addition, infusions with 1L electrolyte solution (Sterofundin ISO B. Braun Vet Care, B. Braun Melsungen AG, Melsungen, Germany) were administered at days 2 and 3.

Blood parameters were checked again at day 3 (Table 1). Hematology showed a PCV of only 0.08 L/L, which had not increased despite the second blood transfusion at day 1. Leucocytosis, lymphopenia, and granulocytosis were still present. This blood sample also revealed an increased level of band neutrophils. Hyocalcemia and hypomagenemia had worsened compared to the first blood sample. Hypoproteinemia, hypoalbuminemia, and hyperbilirubinemia were also still present in the plasma of the goat. In addition, hyponatremia had occurred. The test for melena was also repeated at day 3 and revealed a positive result again.

Due to the still very low PCV, another blood transfusion was performed at day 4. Blood from another donor goat was used and instead of 500 mL, only ~300 mL blood was transfused. For a better assessment of the prognosis of the animal, radiographs of the thorax and abdomen were taken to detect possible metastases of the presumed neoplasia in the abdomen. Clear evidence of metastases could not be found, but the structures in the abdomen were not well-demarcated. Since the animal had not eaten since the previous day and it was recumbent again, the goat was euthanized due to the poor prognosis. A necropsy was performed at the Institute for Pathology, University of Veterinary Medicine Hannover, Foundation.

### Gross Pathology

**Figure 2:**
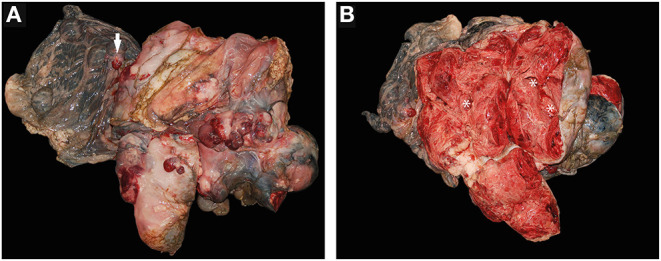
**(A,B)** Abomasal fibrosarcoma from a goat. **(A)** Fibrosarcoma in toto with adjacent mesenterium and mesenterial metastasis (arrow). **(B)** Fibrosarcoma – cross section, showing multiple internal caverns (*) filled with large quantities of seorsanguinous fluids and fibrin accumulations during necropsy.

At necropsy, the animal was in a bad nutritional condition. The subcutis was diffusely edematous. In the pericardium of the animal, 200 mL of a clear, light pink, serous, free fluid was visualized and 1L of serosanguineous, free fluid with fibrin admixtures was found in the abdominal cavity. The pylorus of the abomasum showed a multinodular, cavernous proliferation of 45 x 50 x 40 cm in dimension and multiple adhering duodenal loops, extending from the pyloric wall into the adjacent mesentery (Figure 2A). However, there was no evidence of alterations to the pyloric mucosa. Caverns inside the proliferation were filled with large amounts of serosanguineous fluid and fibrin accumulations (Figure 2B). Two additional round masses measuring up to 3.5 x 4 x 3 cm were found in the neighboring mesenterium at a distance of 10 cm to the main mass. Additionally, multiple nodular, cavernous proliferations of up to 4 x 3 x 1 cm in size were found on the cervix, protruding into the uterine lumen. Further macroscopic findings included a mild to moderate, acute congestive hyperemia of the liver and a moderate, diffuse, acute, alveolar edema of the lungs.

### Histopathological Evaluation

**Figure 3:**
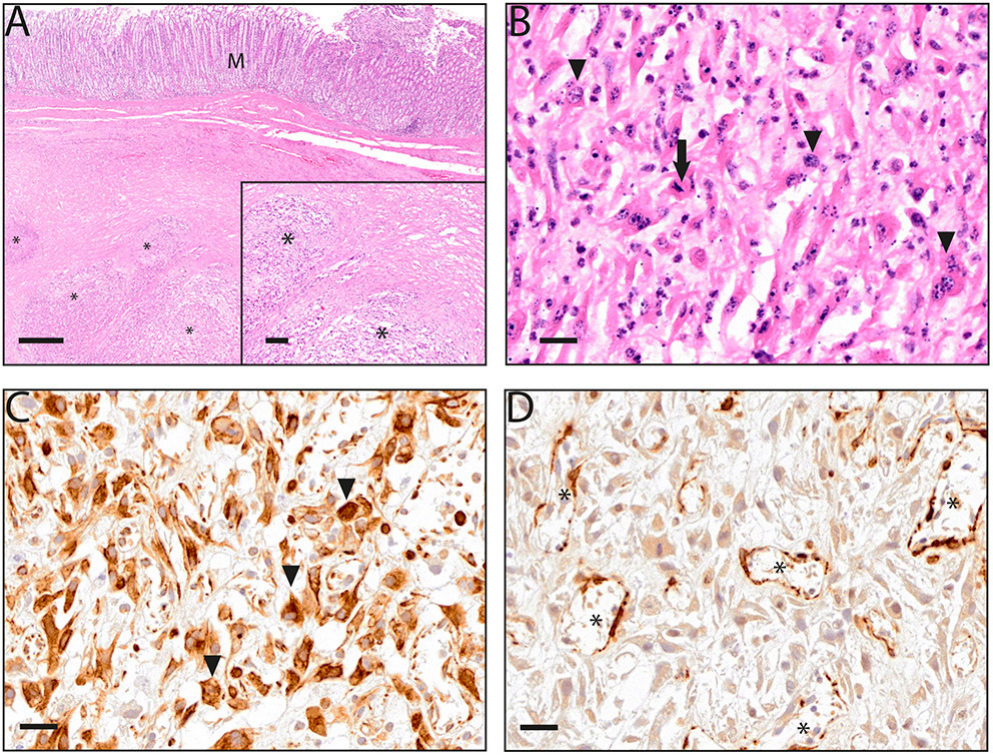
**(A–D)** Microscopic aspects of an abomasal fibrosarcoma from a goat. **(A)** Hematoxylin and eosin staining showing an intact mucosal layer (M) and bundles and streams of neoplastic spindle-cells (*) infiltrating into the tunica muscularis (inlet); **(B)** neoplastic cells showing anisokaryosis and anisocytosis (arrowhead) as well as mitotic figures (arrow); **(C)** highly positive immunohistochemical detection of vimentin in neoplastic cells, immunoreactivity was especially prominent in the cytoplasm of neoplastic cells (arrowheads); **(D)** lack of immunohistochemical detection of smooth muscle actin (SMA) in tumor cells, SMA was detected only in the tumor associated vasculature (*); **(A)** images taken at 10x (overview) and 50x (inlet) magnification, scalebars indicating 500 μm and 200 μm respectively; **(B–D)** images taken at 200x magnification, scalebar indicating 20 μm.

Histological examination of the abomasal proliferation revealed a highly expansive growing, encapsulated, spindle-cell neoplasm. The tumor infiltrated into the *tunica muscularis* from the serosal side and most likely originated from either subserosal, supraserosal or intermuscular stromal tissue. Neoplastic cells were arranged in unorganized bundles and streams supported by moderate amounts of fibrovascular stroma (Figure 3A). The medium sized neoplastic cells were composed of moderate amounts of eosinophilic foamy cytoplasm and indistinct cell boarders. They contained an oval to elongated, medium sized, slightly excentrically located, finley stippled nucleus. Cells were characterized by severe anisokaryosis and anisocytosis, and a mitotic rate of up to two mitoses per high power field was observed (Figure 3B). The tumor showed multifocal extensive areas of necrosis, with associated cavern formation, hemorrhages and, moderate, histiocytic-neutrophilic, resorptive inflammation. The presence of multiple mitotic figures as well as large areas of necrosis were indicative for a malignancy of the neoplasia. Immunohistochemical staining was performed to determine the cell population of origin. Neoplastic cells in the abomasal mass stained positively for vimentin (Figure 3C, a marker of mesenchymal cells) and negative for smooth muscle actin (SMA, Figure 3D, a marker of smooth muscle tissue) as well as tyrosinkinase KIT (KIT, a marker for cells of gastrointestinal stromal tumors, data not shown) and cluster of differentiation 31 (CD31, a marker for endothelial cells, data not shown). This phenotypical characterization of the tumor cells as well as its histopathological features including mitotic figures and necrosis indicated the presence of a fibrosarcoma. The same staining was observed in the adjacent mesenteric masses indicating metastatic spread of the abomasal neoplasia.

The uterine cervix proliferation consisted of a well-differentiated spindle cell proliferation with mild anisokaryosis and anisocytosis, multiple areas of highly variable cell density, which were stained positive for SMA and negative for c-Kit and vimentin and were therefore diagnosed as leiomyoma. The liver showed a centrolobular hepatocellular atrophy and necrosis. Additionally, histological findings included a moderate diffuse vacuolization of hepatocytes interpreted as hepatic lipidosis. Furthermore, a periportal, multifocal, moderate-grade, lymphoplasmacytic hepatitis as well as a mild, multifocal, lymphocytic meningitis of the cerebral cortex were detected.

## Discussion

To the authors' knowledge, this is the first description of severe anemia in a goat and an associated abomasal fibrosarcoma. Fibrosarcomas, malignant tumors of the connective tissue, are characterized by proliferation of fibroblasts. Most fibrosarcomas are observed subcutaneously and cause different symptoms depending on their location (38). A retrospective study published by Löhr in 2013 analyzed data of 1,146 caprine necropsy or biopsy specimens submitted from 1987 through 2011 to the Veterinary Diagnostic Laboratory at Oregon State University, USA (39). In this former study, 100 (8.7%) goats were diagnosed with a total of 102 tumor lesions, of which only two were fibrosarcomas. A 40-year survey carried out by Bastianello et al. did not record any fibrosarcoma in goats (40). A single case report of a fibrosarcoma in the gastrointestinal tract of goats was published by Pesato et al. (41). They described a fibrosarcoma in the rumen of a 9-year-old male dwarf goat, which had developed metastases in the liver. However, the abomasum was not affected in this case. This animal was also anemic, but in contrast to the goat in the present case study, was of a non-regenerative character (41). Other abomasal neoplasias in goats are described by Valedre et al. (42) and Smith et al. (43). Valedre et al. found neoplasias associated with rumen, omasum, and abomasum in two Spanish Ibex. In contrast to the fibrosarcoma described in the present case, the tumors in these cases were positive for KIT and identified as a gastrointestinal stromal tumor (42). Smith et al. found a hamartoma in the pyloric region of an 18-month-old La Mancha wether (43). Similar to the goat in our case, this animal was also described as lethargic and anorectic.

Several other abomasal neoplasia are described for cattle, deer or camelids. In cattle, abomasal tumors are often lymphosarcomas; in 41 per cent of the animals suffering from lymphosarcoma the abomasum was affected (44). In addition, there are reports of abomasal harmatomas, adenomas or adenocarcinomas in cattle (45–47). Abomasal adenocarcinomas have also been described in other species, including elk, guanaco or Arabian camel (48–50). In 2018, Tharwat et al. described the case of a 15-year-old female Arabian camel suffering from an omaso-abomasal adenocarcinoma that was also associated with anorexia and severe anemia, with a PCV of 0.075 L/L similar to the goat described in the present case study (50).

Gastric neoplasia are also frequently associated with anemia in other species. In a study of 24 horses with different gastric neoplasia, Taylor et al. found anemia in 37% of the animals, and concluded that gastric neoplasia should be considered as a differential diagnosis in acute internal hemorrhages (51). Although most of the horses in this former study had squamous cell carcinoma, none with fibrosarcoma, there is also evidence that fibrosarcoma may also be associated with anemia. In 38 human cases of inflammatory fibrosarcoma, in 31 of these mesentery was located and retroperitoneum anemia was reported in 21 of the cases (52). According to Willard, anemia associated with gastrointestinal neoplasia in dogs and cats can be caused by bleeding due to ulceration, or, in chronic cases by iron deficiency (53). In the present goat, both factors seem to play a role. On the one hand, there was blood loss due to the tumor, indicated by the presence of multifocal acute hemorrhages inside its stroma; on the other hand, the iron deficiency indicates a chronic condition. As no signs of damage to the pyloric mucosa were found during the pathological examination of the animal, the observed melena are probably the result of microlesions triggered by an increased consumption of coagulation factors, which would be supported by the thrombocytopenia. The presence of reticulocytes also shows that the character of the anemia was regenerative. The extremely low PCV combined with the presence of melena therefore suggests intestinal blood loss secondary to the abomasal fibrosarcoma. Other causes for anemia seem unlikely in this goat despite the fact that *H. contortus* involvement cannot be completely ruled out due to the low level of gastrointestinal nematodes in the fecal sample. However, this seems unlikely since affected animals typically exhibit massive fecal excretion of Haemonchus eggs. In addition, there was no evidence of liver fluke, hemoparasites or hemolysis. The animal's copper and selenium supply were within the normal range. The role of cobalamin remains unclear, since this was not determined.

The congestive hyperemia observed in the liver as well as the centrolobular, hepatocellular atrophy and necrosis, in conjunction with the serous effusions observed in the abdomen and pericardium, indicate an increase in portal blood pressure caused by the neoplasia. The periportal, lymphoplasmacytic hepatitis and the low-grade, multifocal, lymphocytic meningitis are most likely inflammatory processes of unclear origin, which are independent of each other and of the neoplastic process. The clinical relevance for the present case cannot be conclusively assessed. The increased activity of GLDH as well as the hyperproteinemia and hypoalbuminemia could be associated with disintegration of liver cells. However, it remains unclear why there was a subsequent decrease in GLDH (Table 1: day 3). This could possibly be explained by dilution due to the blood transfusion. Furthermore, the damage to liver cells appears to be limited, as the activities of the liver-specific enzymes ASAT and GGT were not increased.

The leiomyoma detected in the cervix in this case seems to be an independent neoplastic process and was not linked to the present disease of the goat. Cervical leiomyomas occur regularly in older female goats (54).

## Conclusion

In older goats with dysfunction of the abomasum, abomasal fibrosarcoma should also be considered as a differential diagnosis. Gastrointestinal tumors may also lead to anemia due to large intestinal blood losses. Clinical indications may include emaciation, apathy, pale mucous membranes, and the presence of melena.

## Data Availability Statement

The original contributions presented in the study are included in the article, further inquiries can be directed to the corresponding author.

## Ethics Statement

Ethical review and approval was not required for the animal study because all data used for this study were collected during clinical treatment and pathological examination and were obtained to diagnose the clinical case. Written informed consent was obtained from the owners for the participation of their animals in this study.

## Author Contributions

MW and MS diagnosed and treated the clinical case. GB and WB performed the pathological and histological examination. AP and TG performed the clinical laboratory tests. MW wrote the manuscript. GB provided the description, figures, and discussion of pathology and histology. MS provided the clinical case description. The study was designed by MW and GB and supervised by MG and WB. All authors read and approved the final manuscript.

## Funding

This Open Access publication was funded by the Deutsche Forschungsgemeinschaft (DFG, German Research Foundation) within the programme LE 824/10-1 Open Access Publication Costs and University of Veterinary Medicine Hannover, Foundation.

## Conflict of Interest

The authors declare that the research was conducted in the absence of any commercial or financial relationships that could be construed as a potential conflict of interest.

## Publisher's Note

All claims expressed in this article are solely those of the authors and do not necessarily represent those of their affiliated organizations, or those of the publisher, the editors and the reviewers. Any product that may be evaluated in this article, or claim that may be made by its manufacturer, is not guaranteed or endorsed by the publisher.
